# Never Too Much—More talent in football (always) leads to more success

**DOI:** 10.1371/journal.pone.0290147

**Published:** 2024-02-08

**Authors:** Ashley M. Long, Mario Graf, Merim Bilalić

**Affiliations:** 1 Department of Psychology, University of Northumbria at Newcastle, Newcastle upon Tyne, United Kingdom; 2 Department of Psychology, University of Klagenfurt, Klagenfurt am Wörthersee, Austria; Universiti Malaysia Terengganu, MALAYSIA

## Abstract

Though it may appear counterintuitive, certain positive attributes can eventually have negative consequences when taken to an extreme. This concept is exemplified in sports, where an increase in talent among team members initially leads to improved success, but beyond a certain threshold, excessive talent can adversely affect the team. This occurrence is known as the Too Much Talent (TMT) effect, wherein status conflicts among highly skilled players can hinder team performance, particularly in sports that require coordination and cooperation. While early evidence supported the TMT effect in team sports, its validity has recently been challenged. In this study, we analyzed a comprehensive dataset consisting of 780 data points across 42 seasons from seven top European football (soccer) leagues to examine the TMT effect’s presence. Our findings reveal that football does not exhibit the TMT effect. Instead, we observed a consistent, positive correlation between the number of skilled players on a team and team success. Additionally, talent did not display diminishing returns, as its impact on success remained stable even at the highest concentrations of talent. We relate our results to existing theories and propose that future research comparing more individualistic and interdependent team sports could further enhance the field.

## Introduction

In the field of psychology, there exists a phenomenon known as too-much-of-a-good-thing (TMGT), where generally positive traits start to exert negative influence after a certain point (for reviews, see [1, 2]; for a general framework, see [3]). As far back as the 4th century BCE, Aristotle [4] claimed that moderate amounts of qualities, rather than an abundance thereof, are necessary for success. This concept has been studied extensively, with various TMGT phenomena observed across multiple domains, including management and organisational behaviour [2, 3], conscientiousness [5], optimism [6], and expertise [7, 8].

In the realm of sports, a similar phenomenon has been proposed, where having more talented team members leads to better team performance up to a certain point, after which talent becomes "too much" and detrimental to performance [9]. This notion of the too-much-talent (TMT) effect suggests that status conflicts among highly skilled members may impair coordination in teams. While this effect has been observed in sports with high coordination requirements, such as basketball and soccer [9], recent studies have questioned the appropriateness of the approach used to test the inverse-U-shaped relation [10, 11]. To address this question, we conducted a study using a much larger dataset of 780 data points across 42 seasons of the best seven leagues in European football. Our goal was to investigate whether the TMT effect is present in this context using previously established appropriate methods for checking the inverse-U-shaped relations [10, 11].

### Talent in sports

Talent is considered to be one of the most fundamental aspects of all levels of competitive sport when considering performance, development, and potential of both individual athletes and teams [12, 13]. Although a true underlying definition and conceptualisation of what talent is in a sporting context is missing, a general consensus is reached in research, alluding to talent as an ability to demonstrate remarkable ability and achieve peak performance [14, 15].

This then creates the notion that the more talented an individual is within their respective sport, the greater the likelihood of reaching expert performance and achieving sporting success [16]. For example, these individual effects have been shown amongst a plethora of research articles, with both old and new research continually suggesting the importance of talent within sporting success [17–19].

With this clear indication, and supported evidence that talent positively impacts individual sporting success and elite performance [16], this has led to the notion, and typical belief, that the prevalence of top talented individuals within a team will also, undoubtedly, improve team success and performance. Such beliefs have been shown in research depicting that people generally believe that the relationship between top talent within a team, and team performance is linear, indicating that the effect of more top talent can only bring about positive effects, without much diminishing returns [9]. For example, Dirks [20] also found that higher levels of talent within a team was one of the greatest determinants of team success in their respective sample of basketball players. Despite this popular belief, however, it may be possible that the opposite is true, suggesting that there may be such a thing as ‘too much talent’ (TMT) within a team, exerting negative effects on team performance.

### TMT effect

However, in the context of team sports, this suggests that talent within a team may initially employ beneficial effects and facilitate performance, however only up to a certain point. Beyond this point, more talent will eventually start to result in negative effects on team performance, as seen in Swaab et al. [9], causing a TMT effect. When looking at this TMT effect, literature can explain why there can be such a thing as excess talent on a team, and how this may actually employ negative effects. For example, Bendersky and Hays [21] suggest that there may be group conflicts over status. If a team has multiple top talented players, they may compete for dominance, therefore causing disputes within the team surrounding hierarchy and status, resulting in a focus on an individual’s social rank within a team as opposed to focusing on getting good results and coordinating [22]. Recent research also backs up this explanation, suggesting that this conflict creates a”status threat” therefore effecting multiple athlete’s willingness to socialise with teammates, especially newcomers [23]. Therefore, it is suggested that more top talent within a team will result in more disputes over hierarchy and status, resulting in a gradual loss in coordination, impacting overall team performance.

Although research alludes to why TMGT phenomena and TMT effects can likely exist within sport, there is, without question, a large gap in research in regard to specifically analysing this ‘inverted U-Shaped’ relationship between the amount of top talent within a sports team, and team performance. For example, one of the first studies to specifically look at this relationship is the aforementioned work of Swaab et al. [9]. This phenomenon was demonstrated in multiple team sports [9] including basketball and football, with results showing that after a certain point, talent indeed did become too much and consequently exerted negative effects on team performance. Within this study, 30 National Basketball Association (NBA) teams over 10 seasons were analysed, determining team performance as win percentage at the end of the year for each season, and identifying top talent as players whose Estimated Wins Added (EWA) were within the top third of the overall cohort. EWA being a statistic in which portrays a player’s total contribution within the team.

When looking at the variables within the study for football, team performance was determined through FIFA rankings during qualification periods for the 2010 and 2014 World Cups. Top talent was identified through calculating the percentage of players within the roster of each national team who had contracts with elite clubs. Interestingly, this TMT was not found for the sport of baseball, where the coordination requirements are lower and more individual work is required, suggesting less status conflict and therefore less of a negative impact on team performance [9, 10].

The analysis method used within this paper, and therefore considered to be the norm within this domain of research, is quadratic regression. If this analysis demonstrates a linear coefficient in which is positive, illustrating initial development, and a quadratic coefficient in which is negative, this negative quadratic coefficient will progressively force the curve to bend and ultimately trend downwards, therefore demonstrating an inverted U-shape relationship [10, 24]. However, it is also important that the peak of this curve, or inflection point (where positive effects will transform into more detrimental effects) is comfortably within the overall talent range [10].

When being critical of the study, it does demonstrate strengths such as carrying out robustness tests, involving varying the cut-off points for variables such as top talent to test if results could be replicated with different thresholds. However, despite being one of the first studies focusing on the relationship between top talent and team performance, it received significant criticism regarding the analysis method used (quadratic regression). For example, Nelson & Simonsohn [25] and “Should this paper in Psychological Science be retracted?” [26] have since criticised the original work by Swaab et al. [9], explaining how the sole use of quadratic regression proves problematic when used within this form of research. The specific reasoning behind why this analysis method is an issue in this context is because there is a rather large rate of false positive results concluding inverted U-shaped relationships where they do not truly exist [11].

For example, this is often seen when there are minimal data points beyond the peak of the curve, or inflection point, resulting in the analysis method sometimes indicating an inverse U-shaped relationship even when there may not be one [11]. In context, this may have led to the analysis within Swaab et al. [9] showing that too much talent on a team can harm team performance, even when this relationship may not be as strong as quadratic regression would suggest. These criticisms have therefore led to the notion that quadratic regression does not accurately capture the relationship between talent and team performance or success [10].

Resultantly, Gula et al. [10] commented on the Swaab et al. study [9], while also conducting their research on a much larger sample than initially seen in the original work, consisting of 64 NBA seasons, while analysing the data using more suitable analysis methods, providing greater clarity, and helping to bridge the gap in this area of research. Upon rigorous analysis of the larger dataset, the findings of this more recent paper opposed those that were found in Swaab et al. [9], suggesting that the effect of talent on success becomes smaller as the amount of talent increases, but that there is never a negative effect of talent on the success in the NBA. The authors did not only used two lines regression [11] to support their conclusion, but also a modelling approach where several different functions were tried (and most of whose outperform the quadratic one). The authors proposed that instead of a TMGT effect, a Never Too Much (NTM) effect should be considered.

### Current study

As a result of the findings of Gula et al. [10], it is important to expand on this area of research by assessing the relationship between top talent and team performance in further sports, with more informed and appropriate analytical procedures, specifically the modelling approach and the two lines regression method as suggested in prior research [10]. While only two studies have focused on this area, it is still important to draw from both to accurately expand on this domain. Therefore, this study aims to advance this area of research by using more accurate analytical procedures, specifically looking at the relationship between the number of top-talented performers in a team, and team performance within football. It seems logical to use football as the next steppingstone in the expansion of research in this domain as this can, like the research conducted by Gula et al. [10], help to rectify and provide clarity on whether the team sports initially included within the work of Swaab et al. [9] indeed do demonstrate or do not provide evidence for the TMGT phenomena and TMT effects. The findings of Swaab et al. [9] suggested that a TMT effect was prevalent in football, although the subsequent analysis [11] came to a widely different conclusion. A new aspect here is that we focus on football leagues rather than the World Cup qualification periods as in Swaab et al. [9] in this study. This method will result in more data points and therefore provide a greater demonstration of whether a TMT effect persists over a sustained period. It is hypothesised that, in line with the findings of Gula et al. [10] and Simonson et al. [11], a NTM effect will persist within football, showing that a higher prevalence of top talent within a team will not have a deteriorating effect on team performance.

## Method

### Design

This study utilized a correlational design based on secondary data. This design method is suitable for this type of study, as it aims to assess the correlation between two variables, specifically looking for an inverted U-shaped relationship. The dependent variable for the main analysis was team performance, which was measured by the proportion of points won in a season out of all available points in that season. The independent variable was top talent, measured by the number of talented players in the team during that season. The roster size was used as a moderating variable, consistent with previous research [9, 10]. The study received ethical approval from the Northumbria University Psychology Ethics Chair (Submission Reference #47994).

### Measures

#### Team performance

Team performance was calculated as a winning proportion over the course of a season, taking into account the points system in football, which awards 3 points for a win, 1 point for a draw, and 0 points for a loss. Winning proportion was calculated by classifying the value of a draw as a third of a win. Higher values indicate a higher win rate for each team. Data for team performance was retrieved from the statistical website FotMob (https://www.fotmob.com) and checked for consistency with another statistical website, Football Reference (https://www.fbref.com). Six seasons between 2016/17 and 2021/22 were analyzed for the top seven leagues in Europe (https://www.uefa.com/nationalassociations/uefarankings/country/#/yr/2023): Premier League (England), La Liga (Spain), Bundesliga (Germany), Serie A (Italy), Ligue 1 (France), Eredivisie (Netherlands), and Portugal Liga (Portugal). This resulted in 780 data points, comprising six seasons for 130 teams per season.

#### Top talent

To identify top talent players within each team, we followed previous research [9, 10] and calculated the proportion of top talent players in a given league in a given season. FotMob individual player ratings were used as the player performance measure, which is based on a comprehensive dataset from Opta (https://www.statsperform.com/opta-football/). The FotMob rating system covers more than 300 individual stats per player per match across over 375 competitions worldwide, making it a reliable and consistent source of data that captures various aspects of player performance. FotMob rating accounts for advanced statistical models, such as expected goals (xG) and expected goals on target (xGOT), which measure the quality of chances and shots respectively. These models help to account for the randomness and luck factors that affect the outcome of a match, providing a more accurate representation of how well a player performed. FotMob rating provides each player with an overall rating ranging from 0 to 10, with 0 being the lowest rating and 10 being the highest.

To define top talent players within each team, a player was coded as top talent if their player rating for the season was better than a certain percentage of all players in that league in that season. Six different cut-offs were used to establish robustness of findings: better than 65%, 70%, and 75%, as used in previous research, as well as higher thresholds of 80%, 85%, and 90%. The proportion of top talent within a team was calculated by dividing the number of players fitting within the top-talent cut-off points (65%, 70%, 75%, 80%, 85%, and 90%) by the total number of players on the team.

To validate the analysis, we used the WhoScored rating system (www.whoscored.com), which uses a distinctive statistical algorithm to obtain a performance rating for a player based on over 200 in-game indicators. Unlike FotMob, WhoScored rating does not allow automatic web scraping of ratings, so we manually obtained all available individual ratings for the English Premier League and Spanish La Liga. The Premier League WhoScored ratings were obtained from 2009/10 to 2021/22, and the ratings for La Liga were available from 2016–17 to 2021/22. For this additional check, we used the 70% cut-off (see Online Supplementary Material, OSM).

#### Control variables

To ensure the robustness of findings, we used different cut-offs for defining talent. We also controlled for roster size, that is the measure of the total amount of players who played within the team in that season. A player qualifies for the FotMob rating if it has played at least 50% of all matches and at least 90 minutes (for the per 90 stats) to be included in the rankings (https://www.fotmob.com/faq).

### Analysis

We employed two analyses in this study. The first was a modeling approach, as applied in Gula et al. [10]. We fitted a number of functions, including linear (e.g., linear, quadratic, and cubic) as well as non-linear (e.g., logistic, power, and log) functions. We assessed the fit of each function to the data and identified the one that best described the data. We conducted this analysis for all six cut-offs, ranging from 65% to 90% in 5% steps.

The second analysis used the Two Lines Regression [11], which estimates an interrupted regression. This type of regression consists of two individual slopes. If both slopes have opposite signs and are statistically significant, the two-lines test will reject the null hypothesis that no u-shaped effect is present. The “Robin Hood” algorithm is used in this analysis to set the breakpoint between the two slopes, which gives the test more power to determine a u-shaped effect if one is present [11]. The test offers different models that can be used to test for U-shaped effects, both with and without control variables. To perform a more robust analysis, we ran the model yx+x2 to determine whether x2 (the top talent variable) had a U-shaped effect on y (team performance) while controlling for x (roster size). Additional models were run using yx to determine whether top talent had a U-shaped relationship with team performance without the inclusion of the control variable of roster size (see OSM). Before conducting the actual analyses, we performed an outlier analysis, as done in Gula et al. [10], which confirmed that there was no reason to exclude any data points in our dataset.

## Results

### Descriptive analysis

On average, the teams in the sample won 0.47 of possible points (SD = 0.16), and the team size, defined as all players who played over 50% of the season games, each for 90 minutes, was 14 players on average (SD = 1.61). The talent ratio varied depending on the cut-off and ranged from 0.33 to 0.10 for the 65% and 90% cut-offs, respectively (see SOM for detailed descriptive statistics).

Most importantly, team performance was significantly and strongly associated with the talent ratio within the team, ranging from r = 0.88 for the 65% and 75% cut-offs to r = 0.80 for the 90% cut-off. It should be noted that team size had a small positive correlation with team performance (r = 0.20), but the relationship between team size and talent ratio was negligible (r = 0.09). The correlations remained the same once we controlled for team size using partial correlation.

### Modelling and two lines analyses—Talent cut-offs 65%, 70%, and 75%

Previous studies [9, 10] defined top performers (talent) as the top third performers (i.e., players who performed better than 66% of all players). To ensure robustness, we present results for three talent cut-offs: 65%, 70%, and 75%. The results for the more stringent talent cut-offs (80%, 85%, and 90%) are presented in the next section.

**Fig 1:**
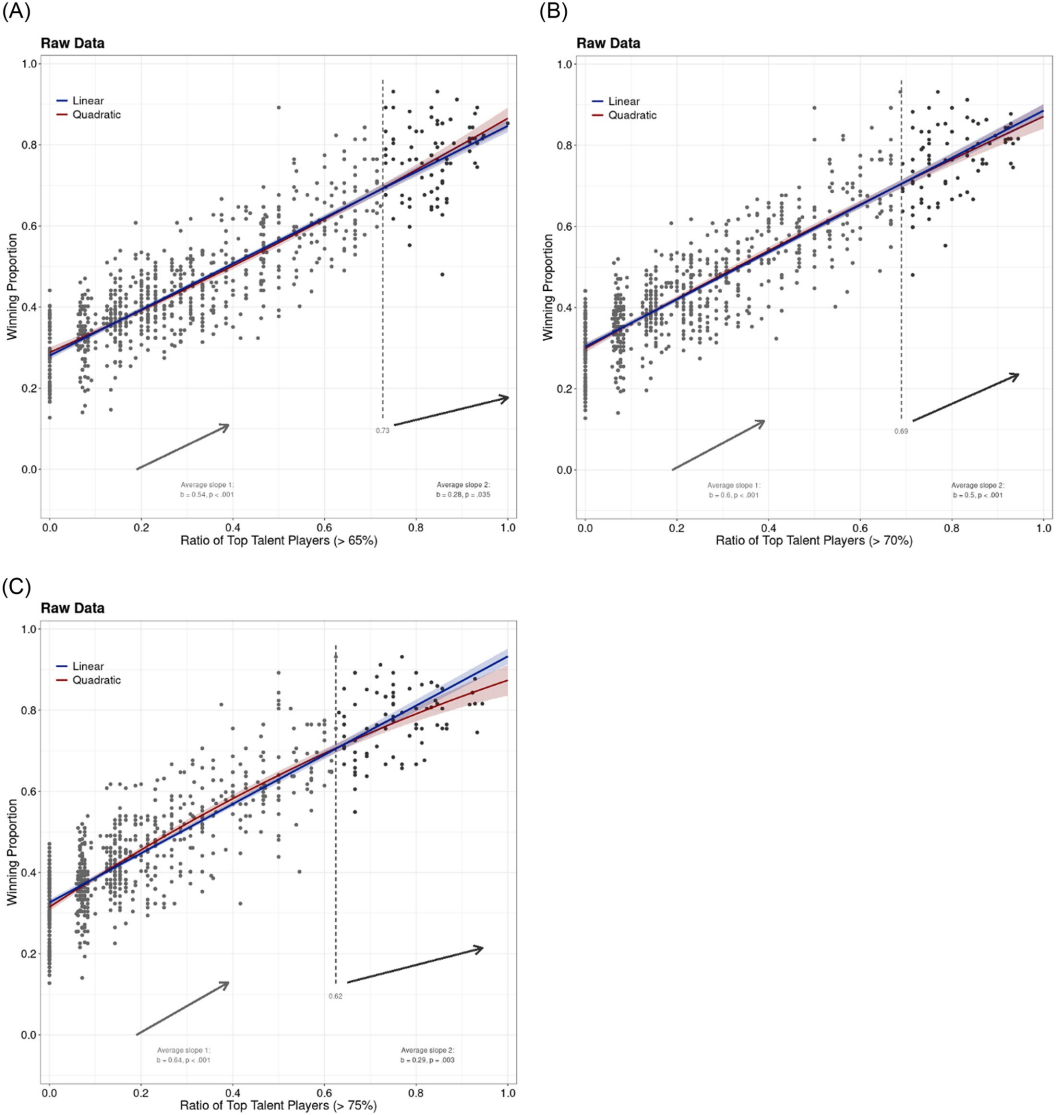
Team success (defined as the winning proportion) as a function of the ratio of top talented players based on FotMob ratings in top European football leagues. The left panel depicts the talent cut-off 65% (i.e. top 35% players), the middle panel talent cut-off 70%, and the right panel talent cut-off 75%. The linear and logistic function fits/lines were plotted. The small red dashed vertical line in between indicates the break point of the quadratic function. The two average slopes of the interrupted regression are also presented below together with coefficients. The gray dashed vertical line in between indicates the break point.

Fig 1 shows the team talent ratio-team success relationship for 65% (left panel), 70% (middle panel), and 75% (right panel) talent cut-offs. In all three cases, the association is straightforward—the more talent on the team, the better the team performs. The quadratic function does not produce a typical decreasing curvilinear pattern that would indicate a TMGT effect, unlike in other studies (e.g., [9, 10]).

This was also evident in our two lines approach [11]. As expected from Fig 1, the analysis shows that the TMGT effect is not present in this case. The break was around.70 (more precisely,.73,.69, and.62 for 65%, 70%, and 75% talent cut-offs, respectively), and there were enough data points beyond the break (see Fig 1). However, the second slope was always increasing and highly significant (b265% = 0.28, p = .035; b270% = 0.50, p < .001; b275% = 0.29, p = .003). These results suggest that performance continually increases as the proportion of top talent increases within a team, without any detrimental effects on performance after a certain threshold of talent is reached. In other words, there is no evidence for a TMGT effect in our data when we used the common cut-offs for talent as employed in previous research [9, 10].

We formally tested this observation by fitting three functions of non-linear family and three of linear (see Table 1). In order to establish how well different functions fit the data, we used log-likelihood value (LogLik), where higher numbers indicate better fits, as well as, Bayesian information criterion (BIC) and Akaike information criterion (AIC). Both BIC and AIC consider the complexity of the model as more complex models inevitably fit data better, but BIC punishes complexity more than AIC. In both cases, lower numbers, including negative values, indicate better fits. Finally, we also use coefficient of determination, R^2^, which indicates the amount of variance explained.

**Table 1:** Goodness of fit for linear and non-linear models for 65%-75% cut-offs.

Function	LogLik	BIC	AIC	df	*R* ^2^
**65% Talent cut-off**					
Logistic	817	-1615	-1628	3	-
Power	549	-1077	-1093	3	-
Log	664	-1308	-1321	3	.65
Linear	877	-1733	-1747	3	.78
Quadratic	878	-1730	-1748	4	.78
Cubic	878	-1723	-1746	5	.78
**70% Talent cut-off**					
Logistic	846	-1672	-1686	3	-
Power	445	-870	-884	3	-
Log	651	-1283	-1297	3	.67
Linear	881	-1743	-1757	3	.78
Quadratic	882	-1738	-1756	4	.78
Cubic	883	-1732	-1755	5	.78
**75% Talent cut-off**					
Logistic	838	-1656	-1670	3	-
Power	323	-626	-640	3	-
Log	623	-1227	-1240	3	.68
Linear	848	-1676	-1690	3	.76
Quadratic	854	-1682	-1701	4	.77
Cubic	855	-1676	-1670	5	.77

Table 1 shows that the linear function is certainly not worse in describing the data than the more complex quadratic or even cubic function. As a matter of fact, the quadratic terms in the quadratic functions is almost non-existent with no inflexion point (when the quadratic function becomes negative, that is starts falling down) within the possible range of results. It should be noted that the fit is rather linear, as there are hardly any diminishing returns on the team performance as the talent ratio increases. This is evident by the low fit indices for non-linear functions (e.g. logistic, log, and power function).

### Modelling and two lines analyses—Cut-offs 65%, 70%, and 75%

**Fig 2:**
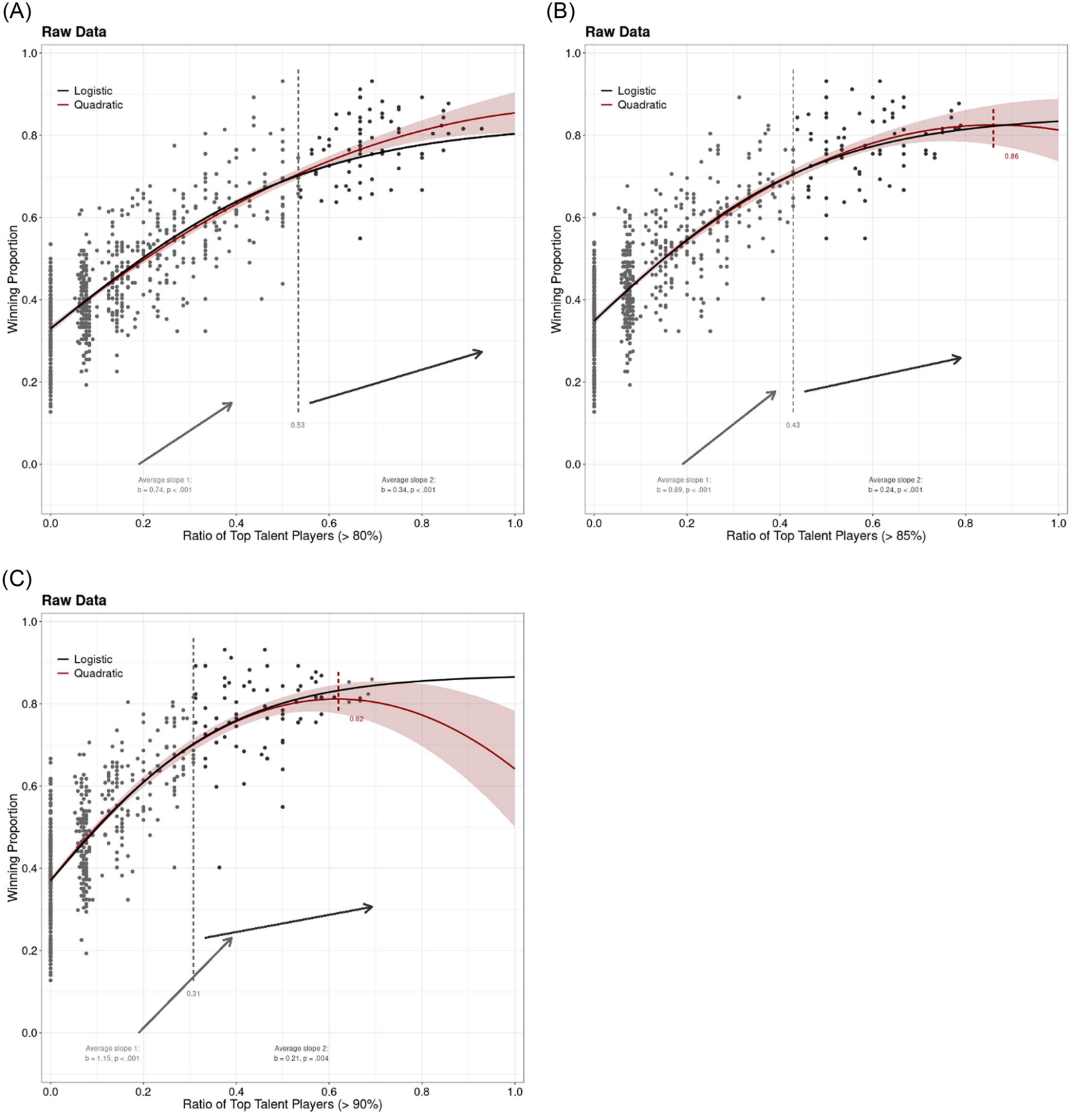
Team success (defined as the winning proportion) as a function of the ratio of top talented players based on FotMob ratings in top European football leagues. The left panel depicts the talent cut-off 80% (i.e. top 20% players), the middle panel talent cut-off 85%, and the right panel talent cut-off 90%. The linear and logistic function fits/lines were plotted. The small red dashed vertical line in between indicates the break point of the quadratic function. The two average slopes of the interrupted regression are also presented below together with coefficients. The gray dashed vertical line in between indicates the break point.

Our next step was to check the relation between talent ratio and team success when we employed more stringent talent cut-offs. Fig 2 plots the data for the team talent ration–team success relation for 80% (Fig 2, left panel), 85% (Fig 2, middle panel), and 90% (Fig 2, right panel) talent cut-offs. In all three cases, the relations were highly positive, but unlike with the lower talent cut-offs, there were some diminishing returns as the talent ratio increased. There no TMT effects, however, as can be seen when the quadratic function was plotted. There was no inflexion point for the 80% talent cut-off, while the break was at.86 talent ratio for the 85% talent cut-off, but there were no data points after this break. The break point for the strictest talent cut-off (90%) is somewhat closer at.63, but there are only six data points after the break, and none of them are indicating a declining relation. This has been confirmed by the two lines analysis, as the second line is never declining. Actually, in all three cases it is increasing and is statistically significant (b2_65%_ = 0.34, p < .035; b2_70%_ = 0.24, p < .001; b2_75%_ = 0.21, p = .004).

As with the lower talent cut-offs, we formally tested the fit of the data three functions of linear family and three functions of non-linear (see Table 2). Unlike in previous analysis, here a non-linear function, logistic, seems to be the best fitting function. This is particularly the case for the 85% and 90% talent cut-offs. The fact that the logistic function is the best fit confirms that there are diminishing returns at the higher talent cut-offs. In other words, the talent ratio produces better team success, but as the talent within the team increases, the team success does not increase as much.

**Table 2:** Goodness of fit for linear and non-linear models for 80%-90% cut-offs.

Function	LogLik	BIC	AIC	df	*R* ^2^
**80% Talent cut-off**					
Logistic	820	-1620	-1634	3	-
Power	178	-337	-351	3	-
Log	563	-1107	-1120	3	.69
Linear	804	-1588	-1602	3	.73
Quadratic	824	-1621	-1639	4	.75
Cubic	824	-1614	-1638	5	.75
**85% Talent cut-off**					
Logistic	792	-1564	-1578	3	-
Power	25	-29	-43	3	-
Log	458	-899	-911	3	.67
Linear	761	-1503	-1517	3	.70
Quadratic	792	-1558	-1576	4	.73
Cubic	792	-1552	-1575	5	.73
**90% Talent cut-off**					
Logistic	729	-1439	-1453	3	-
Power	-132	283	269	3	-
Log	338	-658	-670	3	.64
Linear	690	-1359	-1373	3	.64
Quadratic	729	-1431	-1450	4	.68
Cubic	729	-1425	-1448	5	.68

### Additional analyses

There are two kinds of additional analyses. We first checked whether the team size influences the results: adding the number of members out of which the talent was calculated did not alter the results (see the OSM). The second analysis used a different rating system, WhoScored player rating, to identify top performers. The results based on the new rating were essentially the same as presented in the main text: the relation between talent ration and team success was essentially linear. Not only there were no negative effects of more talent, but there were also no diminishing returns either—the more talent the team assembled, the more success it had.

## Discussion

Overall, the purpose of this study was to examine the relationship between top talent and team performance while controlling for roster size across 42 seasons and 780 data points in seven of the top football leagues in Europe. The results of this study indicate that a TMGT effect was not present at the elite football club level. Instead, the relationship between talent ratio within teams and team success was linear, with an increase in talent leading to an increase in team success. Even when using strict talent cut-offs such as the top 20% to 10% performers, there was no negative effect of talent on success, although there were some diminishing returns at higher levels of talent ratios.

### TMGT effect in football

The findings of this paper suggest the direct opposite effect than what was originally identified and considered in the findings of Swaab et al. [9]. When looking specifically at this paper, it was suggested that a TMT effect does exist within football, providing evidence for the TMGT phenomenon. There are multiple reasons why those findings may not be consistent with what has been found in this current study. One of them is not, however, the differing methods of analysis used as even the use of quadratic function does not provide support for TMT hypothesis (see Fig 1). However, other factors that may have affected the results. For example, sample size was restricted when only considering teams within the 2010 and 2014 World Cup qualification periods, and therefore only considering a fairly low number of games played for each team within the sample. While ranking team performance in terms of FIFA ranking may have been appropriate, basing inferences of such few data points may diminish the reliability of the negative effect beyond the inflection point. Most likely, the findings within Swaab et al. [9] are a method artifact as the two Lines regression approach applied on the data do not show the negative slope of the second lines (see, [25]). Our data, which included multiple seasons instead of isolated qualification periods, most likely present a more suitable test of the TMT effect than the original data in Swaab et al., [9].

The results found within this paper do, however, support the results that were provided in the work of Gula et al. [10]. Their comment and reanalysis on the original sample of Swaab et al. [9] yielded no evidence for any TMT effect within basketball. Gula et al. [10] used more appropriate analyses after criticising the sole use of quadratic regression, including the modelling approach and the two lines regression method, both applied here. The results here are even more drastic as there is not only a negative relation after the inflection points between talent and success, but there are no diminishing effects of talent on success at the lower, common talent cut-offs whatsoever. Only when talent was defined as the top 20% to 10% performers, there were some diminishing returns as found in basketball study by Gula and colleagues [10]. In football, more team talent equals more team success and even at the very high levels of talent, the additional gains in talent result in similar gains in success as at the low levels of talent.

Although it should be acknowledged that when comparing this present study to the work of Gula et al. [10] different sports were analysed, and the methodologies differ greatly in terms of how top talent is quantified between football and basketball. It is important to note that roster size was controlled for in the current study, with varied cut-off points also demonstrating no form of TMT effect on subsequent analyses. As in Gula et al. [10], where similar variables were controlled for within basketball, they yielded only minor changes in results.

This current study contributes to the literature in multiple ways. Specifically, it was important to build on the original work of Swaab et al. [9], as well as develop the knowledge within this area. While the main purpose behind this study was to advance knowledge, a large influencing factor in much of the methodological considerations was following suggestions made by previous authors who have researched in this domain [10]. Such recommendations included the use of modelling and two lines Regression when focusing on TMGT phenomena, in which this study used. Additionally, through gathering more data points, and using robust measures for team performance and the identification of top talent, this allows to give more clarity and begin to answer the question of whether TMGT phenomena persists within team sports, especially when accounting for control variables and varying cut-off points.

### Limitations

When looking at the limitations of the study, there are multiple considerations that could have been taken within the methodology that may have provided a greater depth to the findings. One of them is the use of black box player ratings provided by FotMob. While based on 300 in-game indicators, FotMob rating is not publicly available or transparent about its calculation method or weighting scheme. It is therefore unclear how it is derived and, for example, how it changes over time. We mitigate this shortcoming by using another black box rating by WhoScored, which produced essentially identical results. Similarly, it is in important to keep in mind that we are not interested in the exact player rating, but rather in top performers and their number in each team. It is inherently easier to identify top 30% or so performers than the exact value of every individual performer. Finally, we varied the definition of talent by using six different cut-offs, none of which produce any evidence for the TMGT effect.

This study’s findings when related to the outcomes of Gula et al. [10] may also have implications on other areas of research. As previously stated, the speculated cause of TMGT and TMT effects within the sporting domain is considered to stem from top talented individuals competing for dominance and social hierarchy within teams [21]. More recent research has proposed that this effect is especially prevalent with newcomers on a team [23]. A better understanding of the TMGT effect may come from research delving into whether the competitiveness for dominance is persistent over time or over consecutive seasons within sports, helping to understand whether athletes learn to accept their places within a team and therefore start refocusing on their main priorities of working together to achieve the best outcomes [22].

Further research looking into more interdependent sports may also prove useful to see if there is evidence of TMGT effects in other sports rather than football or basketball. Aligning with the initial implications within the original study of Swaab et al. [9], future research could benefit from comparing more interdependent sports such as hockey with team sports that have a greater emphasis on individual playstyles and performance (cricket). It is important to continue to use analysis methods such as two lines regression, or other methods such as those outlined in prior studies [10]. This will consequently provide more meaningful results.

## Conclusion

Our research provides evidence against the Too Much Talent (TMT) effect in elite European football clubs. The results support a linear correlation between an increase in talent and team success, contrary to prior assumptions of the TMGT phenomenon. We found no inverse effect of talent on success, rather, an escalation in talent consistently led to better team performance. While we use expansive data over 42 seasons and 780 datapoints, one should keep in mind that the player ratings from FotMob our undisclosed, which may potentially affect the precision of top talent identification. Future investigations should use a more in-depth analysis of how the talent-success relationship varies across different sports, particularly comparing more interdependent sports like hockey with ones cantered around individual performance like cricket. Such comparisons, utilizing analytic approaches like interrupted regression, would offer more comprehensive insights into the TMGT phenomenon. Another venue for future research could be the dynamics of team hierarchies and the interplay of top talented individuals, as their adaptation to team structures over time could influence overall performance.
